# The metabolic plasticity of B cells

**DOI:** 10.3389/fmolb.2022.991188

**Published:** 2022-09-23

**Authors:** Yurena Vivas-García, Alejo Efeyan

**Affiliations:** Metabolism and Cell Signalling Laboratory, Spanish National Cancer Research Centre, Madrid, Spain

**Keywords:** metabolic plasticity, B-cell activation, humoral response, glycolysis, Oxphos, anabolism

## Abstract

The humoral response requires rapid growth, biosynthetic capacity, proliferation and differentiation of B cells. These processes involve profound B-cell phenotypic transitions that are coupled to drastic changes in metabolism so as to meet the extremely different energetic requirements as B cells switch from resting to an activated, highly proliferative state and to plasma or memory cell fates. Thus, B cells execute a multi-step, energetically dynamic process of profound metabolic rewiring from low ATP production to transient and large increments of energy expenditure that depend on high uptake and consumption of glucose and fatty acids. Such metabolic plasticity is under tight transcriptional and post-transcriptional regulation. Alterations in B-cell metabolism driven by genetic mutations or by extrinsic insults impair B-cell functions and differentiation and may underlie the anomalous behavior of pathological B cells. Herein, we review molecular switches that control B-cell metabolism and fuel utilization, as well as the emerging awareness of the impact of dynamic metabolic adaptations of B cells throughout the different phases of the humoral response.

## Introduction

Naïve B cells maintain a state of quiescence coupled to an extremely low basal metabolism that is interrupted when antigen encounter triggers a massive uptake of nutrients and biomass accumulation to sustain a sudden burst in proliferation and a several-fold increase in cell size. Different phases of the humoral response, including the Germinal Center (GC) cyclic transitions from cell growth in the light zone to proliferation in the dark zone, the formation of long-lived, resting but alert memory cells and the antibody production and secretion by Plasma Cells (PC), demand the rewiring of metabolic fluxes to match the functions and energetic requirements of the different phenotypic B-cell states (Boothby and Rickert, 2017).

At the molecular level, key regulators of activated B-cell functions exert a parallel control on metabolism. Therefore, alterations in B cells leading to diseases such as autoimmunity, immune deficiency and B-cell lymphomagenesis occur in the context of abnormal B-cell metabolism, as recently reviewed (Boothby and Rickert, 2017; Iperi et al., 2021; Raza and Clarke, 2021; Shiraz et al., 2022). Thus, understanding normal and dysfunctional B-cell metabolism may uncover potential avenues for interventions to harness pathogenic activities of B cells.

An interesting feature of B-cell metabolism is that it is multifaceted and the reprogramming subsequent to activation is not exclusively based on upregulating a specific metabolic route, such glucose uptake and consumption *via* glycolysis (Dufort et al., 2007; Caro-Maldonado et al., 2014). Instead, increased in B-cell metabolism comprise the involvement of multiple metabolic routes.

**FIGURE 1:**
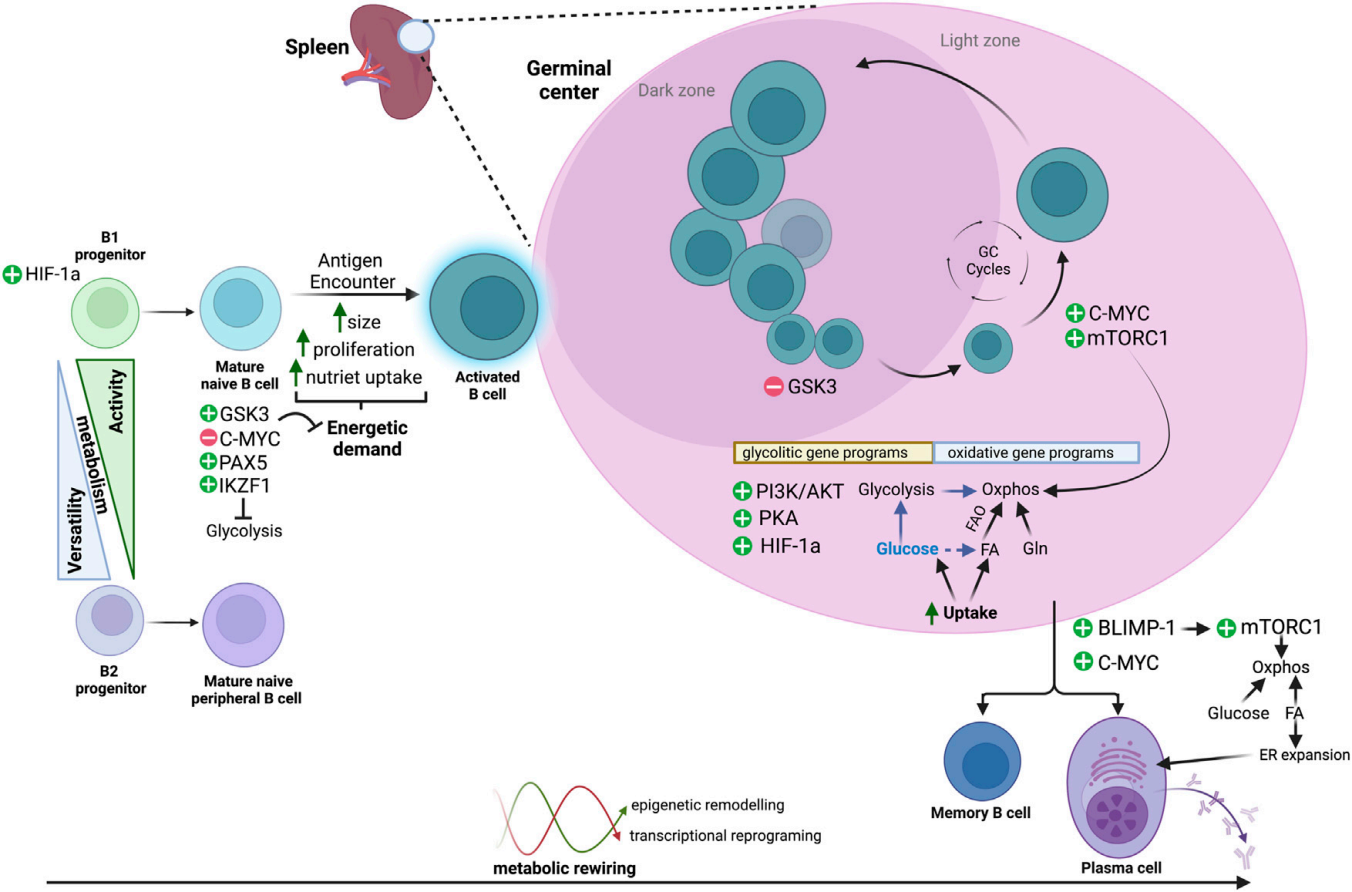
Metabolic routes across B-cell differentiation. B cells display dynamic metabolic changes to meet fluctuating energetic demands as they transition from distinct B-cell lineages, through resting states of low metabolic activity, and following antigen encounter, to an activated and highly proliferative state with increased metabolic activity. B cells activate specific transcriptional programs and regulatory pathways that allow them to either maintain a low metabolic profile or to use a wide range of substrates [glucose, fatty acids (FA) and/or glutamine (Gln)] and different metabolic routes (i.e., glycolysis, Oxphos, FAO). These metabolic transitions are reversible and tightly regulated, and an interplay between epigenetic remodelling and metabolic fluctuations may control transitions throughout the different phenotypic states.

We herein analyze the main metabolic adaptations that occur in B cells through maturation, activation and differentiation, highlighting different metabolic routes, energetic substrates, as well as molecular effectors shaping B-cell metabolism (Figure 1).

## The metabolism of B-cell maturation

B-cell development from lymphoid progenitors derived from hematopoietic stem cells occurs within the fetal liver and bone marrow (BM), and involves subsequent stages of maturation in secondary lymphoid organs such as spleen and lymph nodes. A crucial event in B-cell differentiation is immunoglobulin gene rearrangement, a process that occurs in progenitor pro-B cells and results in precursor pre-B cells that express an immature B-cell receptor (BCR). Further gene rearrangements yield the expression of a mature BCR and the consequent transition to immature B-cell stage. BCR reactivity against self-antigens is then censored *via* apoptosis, while cells expressing non-self-reactive BCRs continue the B-cell maturation process (Martin et al., 2016).

Along with this sequence of events, as early as the B-cell linage is specified, phenotypic heterogeneity goes hand-in-hand with metabolic disparities that will determine distinct B-cell lineages (Sanz et al., 2019; Iperi et al., 2021). For example, B-cell lineages established either from perinatal and peripheral B1 progenitors or from conventional BM B2 progenitors, show distinct fates and metabolic specifications, being tissue-resident B1 B-cells metabolically more active with higher rates of glycolysis, oxidative phosphorylation (Oxphos) and fatty-acid (FA) metabolism, but less versatile than resting follicular (FO) B2 B cells (Clarke et al., 2018).

Beyond lineage-dependent metabolic specificities, pro-B cells in the BM preferentially display a glycolytic metabolism established through the transcriptional control of glycolytic genes in a hypoxia inducible factor 1α (HIF-1α) -dependent manner. Deletion of HIF-1α in mice results in the upregulation of genes involved in Oxphos and the tricarboxylic acid (TCA) cycle, impairing the transition from pro- to pre-B-cell stage (Kojima et al., 2010). Selected transitional B cells continue their maturation process toward resting FO B cells. The phenotypic switch towards mature naïve follicular stages relies on metabolic adjustments that facilitate a quiescence-like metabolic program maintained by the downregulation of Oxphos and inhibition of the mechanistic target of rapamycin complex 1 (mTORC1), in an AMP-activated protein kinase (AMPK)-dependent manner (Farmer et al., 2019).

## Metabolic switches in B-cell activation and the germinal center reaction

The broad repertoire of B-cell phenotypic states is tightly regulated by the combination of specific transcriptional programs and microenvironmental cues that impose metabolic dependencies. The GC are transient micro-anatomical structures that cluster several clones of FO cells that have been activated by a T cell dependent antigen, together with T follicular helper (Tfh) cells and other cell types. Through sequential rounds of Tfh-mediated activation and selection of competing clones of GC B cells, followed by clonal expansion and antibody diversification, the GC reaction facilitates an increase in the affinity of the selected antibody-producing cells, while also yielding memory B cells. Each phase of the GC reaction and the fates of the exiting GC B cells are shaped by profound metabolic changes.

### Glucose and glycolysis: Leading role or supporting actor

Highly proliferative cells increase glucose uptake and its breakdown through glycolysis, even in normoxia, a phenomenon called “Warburg effect” (Warburg, 1956). This metabolic feature, deeply studied in cancer cells, is also relevant during the clonal expansion phase of GC B cells. In this context, the serial reactions of glucose catabolism yield only two ATPs but provide numerous intermediate metabolites (Vander Heiden et al., 2009) used by the growing B cells as bricks for macromolecule synthesis and biomass accumulation.

Before antigen encounter, glucose metabolism is transcriptionally repressed by specific B-cell lineage factors such as PAX5 and IKZF1 (Chan et al., 2017), but following antigen stimulation, B cells maximize glucose uptake through the upregulation of the glucose transporters *GLUT1*, *GLUT4*, *GLUT8* and *GLUT11* (McBrayer et al., 2012), while conclusions on the expression of *GLUT3* are divergent (Doughty et al., 2006; Dufort et al., 2007; McBrayer et al., 2012; Caro-Maldonado et al., 2014). Antigen-mediated induction of glucose transporters is framed within a broader transcriptional reprograming of glycolytic genes (Cho et al., 2016) to guarantee B-cell survival and alleviate the burden of several energetically onerous processes downstream of B-cell activation, such as proliferation and, in case of PC differentiation, to fuel antibody production (Dufort et al., 2007; Caro-Maldonado et al., 2014; Jellusova et al., 2017). These transcriptional changes favor the increase in the glycolytic rate, concomitantly with the activation of pathways that orchestrate B-cell metabolic reprogramming downstream of BCR activation, such as PI3K/Akt signaling (Doughty et al., 2006; Patke et al., 2006; Woodland et al., 2008). In addition, the IL-4/STAT6 axis, with established systemic effects on nutrient uptake and insulin signaling (Ricardo-Gonzalez et al., 2010), also increases glycolysis in activated B cells (Dufort et al., 2007).

The glycogen synthase kinase 3 (GSK3) integrates cytokine and nutrient signaling. GSK3 is a metabolic modulator in resting cells with limited nutrient content and is rapidly inhibited *via* a PI3K/Akt-dependent phosphorylation downstream of BCR and by co-stimulatory signals such as the CD40-CD40L tandem during B-cell-T cell synapse in the GC (Cross et al., 1995). Active GSK3 maintains a low steady-state metabolism in naïve B cells by restraining increments in cell mass and proliferation through the inhibition of the transcription factor C-MYC (Jellusova et al., 2017; Varano et al., 2017). Consequently, GSK3 preserves a restricted energetic status to ensure B-cell survival and allows the early increase in metabolic activity following antigen encounter. B-cell activation results in inhibition of GSK3 by, in addition to the aforementioned PI3K-Akt dependent signals, cAMP- and PKA-dependent stimuli that result in metabolic activation and proliferation (Cato et al., 2011). Importantly, even in conditions of GSK3 inhibition such as after T cell-dependent-stimulation, residual GSK3 activity may be important to harness B-cell proliferation by tuning down glycolysis and mitochondrial biogenesis and to prevent metabolic exhaustion and ROS-mediated cell death (Jellusova et al., 2017; Varano et al., 2017). These antagonistic roles of GSK3, critical for preventing a metabolic collapse of GC B cells, may be conditioned by a dark-zone-to-light-zone decreasing oxygen tension gradient across the GC (Cho et al., 2016). In the normoxic dark zone, higher metabolic rates sustain clonal expansion associated with repressed GSK3 activity while the moderate hypoxic conditions of the light zone contribute to maintain GSK3 activity and its restrictive metabolic effects. Such on-off cycles of GSK3 activity and the repressive effect on C-MYC have been proposed as a main tumor suppressive axis in the GC (Calado et al., 2012; Dominguez-Sola et al., 2012; Finkin et al., 2019).

The interplay of oxygen availability and the cyclic metabolic behavior of GC B cells is mediated by HIF-1α-dependent upregulation of glycolytic gene programs to support anabolism (Cho et al., 2016; Jellusova et al., 2017; Li et al., 2021). It is conceivable that HIF-1α behaves as a metabolic rheostat that adjusts humoral immune responses in a context dependent manner, since both positive and detrimental effects of hypoxia on antibody class-switch recombination have been reported (Abbott et al., 2016; Cho et al., 2016). In contrast to its critical impact during affinity maturation in the GC, the HIF-1α transcriptional program is intriguingly dispensable for the initial glycolytic reprogramming in LPS-activated B cells (Caro-Maldonado et al., 2014).

An emergent concept in GC biology is that the increased metabolic demand coupled to rapid growth and proliferation of GC B cells happens under an environment of transient limitations in nutrients and oxygen (Abbott et al., 2016; Cho et al., 2016), and GC B cells count with an overlapping repertoire of signaling pathways to cope with cyclic metabolic stress. We still need to understand how antibody affinity, metabolic limitations and energetic demands are integrated to maximize the expansion of selected clones without reaching a threshold of deleterious metabolic stress.

The mTORC1 signaling pathway links nutrient availability, anabolism, positive selection and clonal expansion. mTORC1 is involved in GC formation, GC B-cell expansion, metabolic reprogramming and PC differentiation, and both pharmacological and genetic inhibition of mTORC1 largely disrupt antigen-induced cell growth and impair antibody secretion (Cho et al., 2016; Jones et al., 2016; Jellusova et al., 2017; Tsui et al., 2018). The physical interaction of a Tfh and a GC B cell in the light zone is proportional to the amount of antigen presented by the B cell and thus, to the affinity of the antibody expressed. This interaction, called “synapse”, results in rapid activation of mTORC1 and ultimately, in positive selection of the interacting B cell (Ersching et al., 2017). mTORC1 activation in light zone B cells is critical for ribosome biogenesis and anabolic cell growth, processes that precede the proliferative burst that takes place only after B cells have migrated to the dark zone (Victora and Nussenzweig, 2022) (Figure 1). Importantly, mTORC1 activity is strictly required to trigger cell growth but is largely dispensable after activated B cells increase in size and start to execute rapid rounds of cell division (Ersching et al., 2017). The inability to shut off mTORC1 activity by means of constitutively-active nutrient or growth factor signaling to mTORC1 is detrimental and decreases fitness of GC B cells (Goldfinger et al., 2011; Ersching et al., 2017; Long et al., 2022). Interestingly, a moderate, partial increase in nutrient-Rag GTPase signaling results in enhanced B-cell activation, enlarged GC and increased high-affinity antibody production (Ortega-Molina et al., 2019). Moreover, moderately activating mutations in RagC have been exclusively found in human follicular lymphoma (FL) and diffuse large B-cell lymphoma (DLBCL) samples (Green et al., 2015; Okosun et al., 2016), while a mild inhibition of nutrient signaling to mTORC1 impairs the GC response (Ortega-Molina et al., 2021). In addition to its function controlling B-cell growth and proliferation, mTORC1 plays a key role in regulating antibody class-switch recombination, a process that is also tuned by nutrient availability and oxygen tension (Keating et al., 2013; Zhang et al., 2013; Cho et al., 2016). Collectively, these results strongly suggest the existence of exquisitely refined sensitivity of B cells to moderate fluctuations in the nutrient signaling pathway. Finally, beyond the control of the GC reaction, activation of mTORC1 also fuels the rapid extra-follicular proliferation of activated B cells, and this effect is independent from the differentiation into antibody-secreting cells, suggesting that B-cell differentiation and cell division can be uncoupled (Gaudette et al., 2021).

### Oxidative metabolism in B cells: Beyond mitochondrial oxidation of glucose

Despite the relevance of a dynamic control of mTORC1 in B-cell functions, this pathway is not directly involved in the regulation of glycolysis upon antigen-stimulation (Doughty et al., 2006), suggesting that pathways other than glycolysis support the remodeling that allows B-cell differentiation and function.

Increased glucose uptake following antigen stimulation is not merely purposed to feed glycolytic flux, but it also yields glucose-derived pyruvate, ultimately shunted to the mitochondria for an oxidative process that is further supported by an increase in mitochondrial mass (Jang et al., 2015; Jellusova et al., 2017) and by the upregulation of gene programs involved in mitochondrial respiration. Such parallel increase in Oxphos sustains the energetic costs of several consecutive rounds of proliferation during clonal expansion and it is also critical to support PC differentiation by alleviating the energetic burden imposed by massive antibody secretion (Adams et al., 2016; Cho et al., 2016; Price et al., 2018). This increased metabolic capacity of PC is driven by the expression of specific lineage transcriptions factors, such as *BLIMP1*, required for PC differentiation (Price et al., 2018) and the activation of anabolic factors that positively regulate Oxphos by driving mitochondrial remodeling, such as the aforementioned mTORC1 and C-MYC (Tsui et al., 2018).

Glucose-derived pyruvate substantially feeds Oxphos, but other substrates also contribute to fuel respiration. For example, while hampering glutamine metabolism has minimal impact on FO B cells (Choi et al., 2018), glutamine consumption *via* Oxphos is largely increased and is required for GC B-cell proliferation and for PC cells (Garcia-Manteiga et al., 2011; Caro-Maldonado et al., 2014; Waters et al., 2018) highlighting the existence of multiple parallel fuel dependencies in active B cells. In addition to glycolysis and glutaminolysis, recent evidence shows that FA oxidation (FAO) may be a main energetic source for GC B cells (Weisel et al., 2020) in contrast to previous studies pointing to reduced lipid oxidation and increased pyruvate oxidation in B cells stimulated *in vitro* (Caro-Maldonado et al., 2014). The use of lipids as fuel in GC B cells involves mitochondrial and peroxisomal FAO, being both endogenous and exogenously uptaken FA burned preferentially over glucose (Weisel et al., 2020). This FAO-centric vision of the GC is in agreement with the simultaneous transcriptional selective upregulation of genes related with FAO and lipid metabolism in GC B cells (Cho et al., 2016). However, the energetic dependence on exogenous FA has been observed in intestinal PC (Kunisawa et al., 2015; Kim et al., 2016) and B1 B cells (Clarke et al., 2018; Muri et al., 2019), suggesting that lipid metabolism may be relevant beyond GC B cells during the humoral response.

Interestingly, in an apparent contradiction to the increased catabolism, *de novo* synthesis of FA from glucose also constitutes a metabolic adaptation essential to engage PC differentiation (Dufort et al., 2014). Concomitant synthesis and breakdown of FA may be perceived as a futile cycle, but simultaneous catabolism and anabolism of the same substrates is not uncommon in highly proliferative cells such as cancer cells and may enable the profound structural changes that B cells undertake during their PC differentiation process. PC must expand the endoplasmic reticulum (ER) and Golgi apparatus in order to boost the synthesis (and secretion) of antibodies, which may account for 90% of total protein translation in an extremely energetically onerous process. To sustain such biosynthetic and secretory capacity, PC must largely expand the endomembrane compartment, while simultaneously produce large quantities of ATP from FAO, so FA are acquired from external sources and endogenously produced to fulfill both structural and metabolic fates of FA. Of note, the transcriptional regulator of PC lineage differentiation is *BLIMP1*, positive regulator of mTORC1 activity and critical mediator of the Unfolded Protein Response (UPR) (Tellier et al., 2016). Activation of mTORC1 boosts mRNA translation but can also induce proteostatic stress (Goldfinger et al., 2011). In turn, Blimp1-mediated control of the UPR helps counteract the stress resulting from increased translation through the expansion of the ER and Golgi apparatus and thus, the biosynthetic capacity of the antibody-producing cell. Moreover, a link between the UPR effector *ATF4*, amino acid metabolism and autophagy has been recently established in lymphoma cells, suggesting the existence of a functional regulatory connection between UPR and mTORC1 signaling in B cells (Li et al., 2022).

Cancer research has established a solid link between metabolism and epigenetic control of transcription and this concept is emerging in B-cell physiology. Metabolism not only supplies energy and anabolic bricks to support structural remodeling during B-cell differentiation, but also impacts on the epigenetic regulation of specific transcriptional programs. Thus, metabolic rewiring controls B-cell functions and fate decisions by changing the availability of metabolites such as the TCA cycle intermediate α-ketoglutarate, cofactor of the UTX demethylase that regulates expression of the master regulator for GC B-cell differentiation *BCL6* (Haniuda et al., 2020). The phenotypic plasticity that B cells show throughout the GC reaction and the rapid transitions through changes in the expression of specific gene programs are achieved *via* reversible epigenetic remodeling of specific histone marks. These modifications are frequently anomalous in DLBCL and FL due to mutations in genes encoding histone remodelers such as *EZH2*, *CREBBP, EP300 or KMT2D* (Green, 2018; Pasqualucci and Dalla-Favera, 2018; Schmitz et al., 2018; Mondello et al., 2022). Moreover, serine-glycine metabolism cooperates with *BCL2* driving lymphomagenesis through epigenetic silencing of tumour suppressor genes (Parsa et al., 2020). Latent epigenetic differences exist in resting naïve B cells of lupus patients, involving differential expression of metabolic gene sets with roles in Oxphos, UPR and the response to hypoxia (Scharer et al., 2019). Short-chain FA derived from gut microbiota do not merely act as energetic substrates in B cells, but as HDAC inhibitors, thus disrupting antibody responses in healthy mice and murine models of lupus (Sanchez et al., 2020). Thus, awareness of the functional impact of B-cell metabolism as a modulator of epigenetic alterations during lymphomagenesis and in other pathogenic B cells is beginning to emerge.

## Concluding remarks

Throughout each transition in the entire multifaceted lifespan of a B cell, profound metabolic rearrangements occur (Figure 1). We currently count with a limited number of photograms of B-cell metabolism, so the plot of metabolic control of the humoral response remains to be revealed. We need to deepen our understanding on how metabolism is integrated with increasing antibody affinity during clonal evolution, on how conflicting signals are resolved and to what extent does B-cell metabolism dictate the cyclic behavior on the GC. GC B cells show profuse synaptic interactions with the GC microenvironment, but metabolic cross-talk of B cells and other components of the GC remain poorly understood. We also need to learn how aberrant metabolism of B cells precipitates self-reactivity or B-cell transformation. In this regard, deregulated activity of epigenetic modifiers is a hallmark of B-cell lymphomas and our awareness of metabolite availability as a key layer of control for epigenetic modifiers is emerging. The inability to recapitulate the cellular interactions of the humoral response in culture has undermined our insight on B-cell physiology. However, recent technological advances in single-cell technologies, high-content *in vivo* microscopy and in temporal and spatial tracing of metabolites will enable a better understanding of B-cell metabolism and continue settling the perception of its determinant role in dictating B-cell behavior.
